# Optimization of Extrusion Variables and Maleic Anhydride Content on Biopolymer Blends Based on Poly(hydroxybutyrate-*co*-hydroxyvalerate)/Poly(vinyl acetate) with Tapioca Starch

**DOI:** 10.3390/polym10080827

**Published:** 2018-07-26

**Authors:** Chia-Yang Wu, Wai-Bun Lui, Jinchyau Peng

**Affiliations:** 1 Department of Bio-Industrial Mechatronics Engineering, National Chung Hsing University, Taichung 402, Taiwan; joeupking@hotmail.com; 2 Department of English, National Pei-Kang Agricultural and Industrial Vocational High School, Yunlin 651, Taiwan; wblui@pkvs.ylc.edu.tw

**Keywords:** melt extrusion, polymer blends, response surface methodology, optimization, morphology

## Abstract

Poly(3-hyroxybutyrate-*co*-3-hydroxyvalerate) (PHBV), poly(vinyl acetate) (PVAc), and tapioca starch are environment-friendly materials. The present study used these materials to produce biodegradable plastic pellets by melt extrusion. The tapioca starch content of composite formulations, the maleic anhydride content, and the screw speed of the extruder were chosen as variables for the extrusion process. A Box-Behnken response surface design was used to establish mathematical models to predict the relationship between the operating variables and the objective attributes (tensile strength, elongation at break, and water absorption) of the blends. Blend morphology was also assessed. The regression coefficients revealed that the extrusion parameters most significantly affecting extrudate responses were tapioca starch content and maleic anhydride content, both showing significant (*p* < 0.01) linear effects. The results of the analysis of variance found the models are in good agreement with experimental results as informed by high correlation coefficients (*R*^2^ > 0.9), with no significant lack of fit. From the numerical analysis, optimized operating variables (20.13% tapioca starch content, 10.14% maleic anhydride content, and a screw speed of 41.3 rpm) produced a product with optimum values of 16.4 MPa tensile strength, 13.2% elongation at break, and 30.94% water absorption.

## 1. Introduction

Synthetic plastic waste can take thousands or millions of years to fully degrade, and is thus a significant source of environmental pollution, and also poses a serious waste disposal management problem [1,2,3]. Widespread use of plastics disrupts ecosystems and their disposal can contribute to greenhouse gas emissions [4]. Biodegradable plastics (BPs) are renewable and can be degraded naturally by microorganisms from bacteria, yeast, and fungi [5]. Bio-based and biodegradable plastics are derived from natural and renewable resources, including polylactic acid (PLA) [6,7], polyhydroxyalkanoates (PHAs, e.g., polyhydroxybutyrate (PHB) and its copolymer with poly-beta-hydroxy-valerate (PHV) to make (P(HB-*co*-HV)) [8,9], thermoplastic starch [10,11], plastics based on proteins [12,13], and cellulose and its derivatives [14]. Bio-based plastics have attracted increased interest as a potential solution to the problems created by the use of petroleum-derived plastics [15].

Polymer blending, a well-used mixture process by melt-kneading, is a relatively simple and economically viable technique to improve the quality of polymeric materials [16]. As compared to polyolefin, poly(3-hyroxybutyrate-*co*-3-hydroxyvalerate) (PHBV) can be processed for extrusion, injection molding, and blown-film manufacturing, thus decreasing cost of production, low thermal degradation temperatures, and poor melt elasticity [17,18]. Several methods have been developed to improve PHBV properties, processability, and crystallization behavior through blending with poly(ε-caprolactone) (PCL) [19], poly(butylenesuccinate) (PBS) [20], poly(butylene-adipate-*co*-terephthalate) (PBAT) [21], PLA [22,23], and starch [24]. In addition, starch plasticization enhances the interfacial affinity of materials such as glycerol [10,11]. Furthermore, blending polymers with low concentrations of poly(vinyl acetate) (PVAc) may enhance their physical properties [25,26]. To enhance interfacial adhesion between the biofiller and matrix polymer, some studies have sought to melt-blend biopolymers and natural starch by adding maleic anhydride (MA)-modified polymers as a compatibilizer, which grafts onto the polymer backbone to act as a chemical link [27,28,29]. The hydroxyls of starch could react with the anhydride group of MA by ester linkages [30]. The carboxylic groups emerging from the hydrolyzed anhydride could also react with the hydroxyl groups of PHBV by forming hydrogen bonding between carbonyl groups from PHBV and hydroxyl groups from starch [31]. Compared to the chemical blending process, the extrusion as a reactor combines several chemical process operations into a single process without the need of solvent [32]. However, few studies have examined combinations including PHBV, PVAc, and starch during direct extrusion with MA. It is also important to quickly optimize operating conditions and minimize the number of required trials.

Response surface methodology (RSM) combines mathematical and statistical techniques to analyze, model, and optimize processes. One of the most important advantages of RSM is it can help minimize the number of trials required, thus reducing experimental time and costs. In addition, it helps in understanding the interaction between different key variables. Central composite design (CCD) and Box-Behnken design (BBD) are two major approaches to RSM design for fitting second-order polynomials. They are two-level factorial designs that have been modified by adding center points to generate three-level designs and to estimate variability. Compared to the CCD in cases involving 3 or 4 factors, BBD presents some advantages, such as requiring fewer experimental runs and increased efficiency [33]. In addition, BBD is a spherical design with all points lying on a sphere of a $\sqrt{2}$ radius without points at the vertices of the cubic region. This could be advantageous when the factor-level combination points on the corners of the cube are subject to physical process constraints, such as unacceptable operating limits, cost prohibitiveness, or difficulty testing. However, second-order response surface models may not be appropriate for highly nonlinear problems. Design of Experiments (DoE) is used to understand the relationship of operating variables affecting the functional properties of the product, and interactions between different process parameters [34,35]. The RSM approach has been not only applied in extruded plastic product development to determine optimal extrusion conditions, but has also been used to quantify the influence of independent variables on the responses via a process combination [36,37,38].

For the reasons outlined above, we hypothesize that the interactions between variables have a significant effect on the physical properties of blends. Thus, our overall objective is to use response surface methodology to seek the optimum operating conditions of the extrusion process variables (i.e., screw speed), as well as the effects of compositional variables (e.g., tapioca starch and MA contents) on the characteristics of PHBV/PVAc/tapioca starch blends.

## 2. Materials and Methods

### 2.1. Materials

Poly(3-hydroxybutyrate-*co*-4-hydroxyvalerate) with beta-hydroxyvalerate (HV) content of 5% (Y1000, purity of 98.8%) was obtained from Tianan Biologic Material Co., Ltd. (Ningbo, China). PVAc (BP-17s, partial alkalization PVA, PDI of 1.7, *M*_w_ of 84,000–89,000 according to manufacturer’s instructions), an amorphous polymer, was obtained from Chang Chun Petroleum Chemistry Co. Ltd. (Taipei, Taiwan). Tapioca starch (white powder containing 20 wt % amylose and 80 wt % amylopectin) was obtained from Hong Chi Co., Ltd. (Taipei, Taiwan). Glycerol (AR grade) and triethyl citrate (TEC) obtained from Sigma-Aldrich Co. (Taichung, Taiwan) were used as biodegradable plasticizers for PHBV, allowing them to act as internal plasticizers if grafted to the starch backbone or as monomers in repolymerization. MA, obtained from Sigma-Aldrich Co. (Taichung, Taiwan), helps promote bonding between the polyester and the natural starch via maleation. The enzyme solution (analytical reagent grade, obtained from Sigma-Aldrich Co. (Taichung, Taiwan)) had an activity of 0.35 mg/unit per min at pH 6.9 and 20 °C of soluble starch. The amylase obtained from human saliva (Sigma reference A0521) was at a concentration similar to that found in human blood plasma (50 U/L). 1 mL of calcium chloride was used to stabilize the solution.

### 2.2. Sample Preparation and Characterization

Single-screw extruders with simple conveying screws were built to run blends composed by PHBV, PVAc, and tapioca starch. The PHBV/PVAc/tapioca starch blends were mixed with the additives in a 75–25 wt % proportion (i.e., 750 and 250 g). In each treatment (1 kg), the fixed conditions were 91 g of glycerol and 56 g of TEC. The plasticizer TEC was first dispersed in PHBV powder with stirring (K5SS, KitchenAid, St. Joseph, MI, USA) for 1 h, and then mixed well with PVAc at a ratio of 68:32 afterwards. As PHBV/PVAc/tapioca starch blends were set as 100 wt %, PHBV/PVAc blends included various percentages of the mixture of glycerol plasticized tapioca starch (10%, 20%, and 30%, i.e., 75 g, 150 g, 225 g) by weight. As the whole additives were set as 100 wt %, various MA content levels (10%, 14%, and 18%, i.e., 25 g, 35 g, 45 g) were added in the PHBV/PVAc/tapioca starch blends. All mixtures were then stirred for 3 h prior to melting. Before melt-blending, the samples were predried at 50 °C overnight to prevent hydrolysis.

A single-screw extruder with a screw compression ratio of 2.8, a screw diameter of 25 mm, and a length-to-diameter ratio of 32 (YJ-PS25, Yea Jing Machinery Co., Ltd., New Taipei, Taiwan) was used. The rotating screws force the feed material forward towards the die. The cylinder-shaped extrudates were continuously formed from a single circular die hole 2.5 mm in diameter. Compared to a twin-screw extruder, the single-screw extruder offers advantages in terms of cost and design simplicity. Four electrical temperature units operate in the PID control connected to heating/cooling elements on the extruder barrel. A twin-screw volumetric feeder fed the formulas. The experiments were ran at barrel temperatures of 160, 160, 165, and 165 °C with a feed rate of 25 g/min.

The mechanical properties of the specimens were subjected to a tensile test in compliance with ISO 527 Type 5A. After compression molding, specimens were left to stand at room temperature (25 °C) for 48 h prior to mechanical testing. The characterizations were performed using an Instron 4464 mechanical testing instrument (Instron Corp., Norwood, MA, USA) at 5 mm/min and a gauge length of 20 mm between the clamps. The maximum tensile stress (tensile strength) is the stress that a material can sustain without fracture, and is calculated by dividing the maximum load applied during the tensile test by the original cross-sectional area of the sample. The tests were carried out at least five times for each specimen and the obtained data were averaged arithmetically. Water absorption (Y3) was measured using a technique described by ASTM D570 for plastics. The rod-shaped specimens were immersed in distilled water for 24 h, taken out, wiped with tissue paper to remove surface water before weighing, and then remeasured (accurate to only 1/1000). Five test specimens were tested, with the average taken as the result. The micrographic properties were analyzed using a field-emission SEM (JEOL JSM-7401, Tokyo, Japan). The approximate thickness of each sample was 2 mm. The cross-section of samples was held in place by conductive carbon tape on a cylindrical specimen holder, then sputter-coated with pure platinum. The fracture surfaces of the samples were examined at 3.0 KV accelerating voltage and 1000× magnification.

### 2.3. Experimental Design and Data Analysis

Response surface methodology was used with a BBD to depict the effect of the operating variables, including Tapioca starch content (*X*_1_), MA content (*X*_2_), and screw speed level (*X*_3_). Table 1 shows the complete experimental design with three factors at three levels through 15 runs, including 3 central points for experimental factor levels, which contain code and actual values. Experiments were randomized. Multiple responses (tensile strength (*Y*_1_), elongation at break (*Y*_2_), and water absorption (*Y*_3_)) are investigated simultaneously and are thought to be functionally related to the optimal conditions. The study attempts to fit the multiple-regression equations used to describe response quality. The response function (*Y*) is divided into linear, quadratic, and interactive components and is assumed to be fitted to a full second-order regression equation (Equation (1)) [36,37,38].

(1) $$Y=b_{0}+\sum_{i=1}^{3}b_{i}X_{i}+\sum_{i\neq j=1}^{3}b_{ij}X_{i}X_{j}+\sum_{i=1}^{3}b_{ii}X_{i}^{2}\;$$

where *Y* represents the responses (dependent variables), *b*_0_ was the value of the fitted response at the center point of the design (i.e., point (0, 0, 0)), while *b_i_*, *b_ij_*, and *b_ii_* are respectively the linear coefficients, interaction coefficients, and quadratic coefficients.

Experimental results were subjected to statistical analysis using Design Expert version 7 (Stat-Ease Inc., Minneapolis, MN, USA). The quality of the developed models was determined by the *R*^2^ (coefficients of determination) and adjusted *R*^2^. Analysis of variance (ANOVA) was used to validate model significance. To examine the model’s goodness of fit, each term was tested statistically, with each term having F-values of *p* ≤ 0.05. Optimum response values and the expected experimental variable ranges were determined by graphical and numerical optimization techniques. Verified optimal conditions and predicted values were confirmed in triplicate.

## 3. Results and Discussion

### 3.1. Model Fitting

Fifteen observed responses (Table 2) were used to compute the model using the least squares method. Regression analyses for different models indicated that the fitted quadratic regression models accounted for more than 90% of the variations in the experimental data, and the lack of fit of the regression model was not significant.

Multiple regression equations were generated relating tensile strength (*Y*_1_), elongation at break (*Y*_2_), and water absorption (*Y*_3_) to coded levels of the variables. From the experimental data, the developed models were indicated as follows:

(2) $$\begin{array}{l} \text{tensile strength }(Y_{1})\\ =15.83-0.55X_{1}-2.03X_{2}+0.40X_{3}-0.12X_{1}X_{2}-0.23X_{1}X_{3}+0.17X_{2}X_{3}+0.07X_{1}^{2}+0.07X_{2}^{2}+0.22X_{3}^{2}\\ \left(\mathrm{df}=14,\;R_{adj}^{2}=0.997\right) \end{array}$$

(3) $$\begin{array}{l} \text{elongation at break }(Y_{2})\\ =13.00-1.88X_{1}-1.61X_{2}+0.36X_{3}-0.28X_{1}X_{2}+0.23X_{1}X_{3}-0.65X_{2}X_{3}-0.03X_{1}^{2}-1.85X_{2}^{2}-0.75X_{3}^{2}\\ \left(\mathrm{df}\;=\;14,\;R_{adj}^{2}=0.984\right) \end{array}$$

(4) $$\begin{array}{l} \text{water absorption }(Y_{3})\\ =31.17+4.76X_{1}+2.86X_{2}+0.21X_{3}-2.82X_{1}X_{2}-0.40X_{1}X_{3}+0.49X_{2}X_{3}-2.40X_{1}^{2}-1.34X_{2}^{2}-0.33X_{3}^{2}\\ \left(\mathrm{df}\;=\;14,\;R_{adj}^{2}=\;0.931\right) \end{array}$$

Once a model was selected, an analysis of variance was calculated to assess how well the model represents the data. Table 3 shows the ANOVA results for each term of the quadratic model, including linear and quadratic terms, and interaction of effects. A positive sign in front of the coefficient indicates a synergistic effect, whereas a negative sign indicates a negative effect [39]. Generally, *p*-values lower than 0.01 indicate that the model is considered to be statistically significant at the 99% confidence level. Values greater than 0.05 indicate the model terms are insignificant. The adjusted *R*^2^ for tensile strength (*Y*_1_), elongation at break (*Y*_2_), and water absorption (*Y*_3_) (*R*^2^’s = 0.998, 0. 986, and 0.931, respectively) were very high for a response surface. *p*-values for the lack of fit in Table 2 were nonsignificant (*p* > 0.05) thereby confirming the validity of the models, implying all the developed models adequately described the data. The coefficient of variation (CV) indicates the relative dispersion of the experimental data from the model prediction. In general, the CV should not exceed 5% and a smaller CV value implies better reproducibility [40]. In this study, the CV values for tensile strength, elongation at break, and water absorption were all less than 5% (0.507%, 2.22%, and 4.293%, respectively). Interactions between variables with a significant effect on responses are shown in Table 2. Instead of studying single variables (as in the conventional method) the interactions are investigated, which is significant and important for a comprehensive optimization study. Insignificant lack of fit of the three responses is a good result as the primary objective was for the model to fit the experimental data. The 3D surface response and 2D contour plots are provided as graphical representations of the regression equation used to visualize the relationship between the response and experimental levels of each variable. 

### 3.2. Tensile Strength

**Figure 1:**
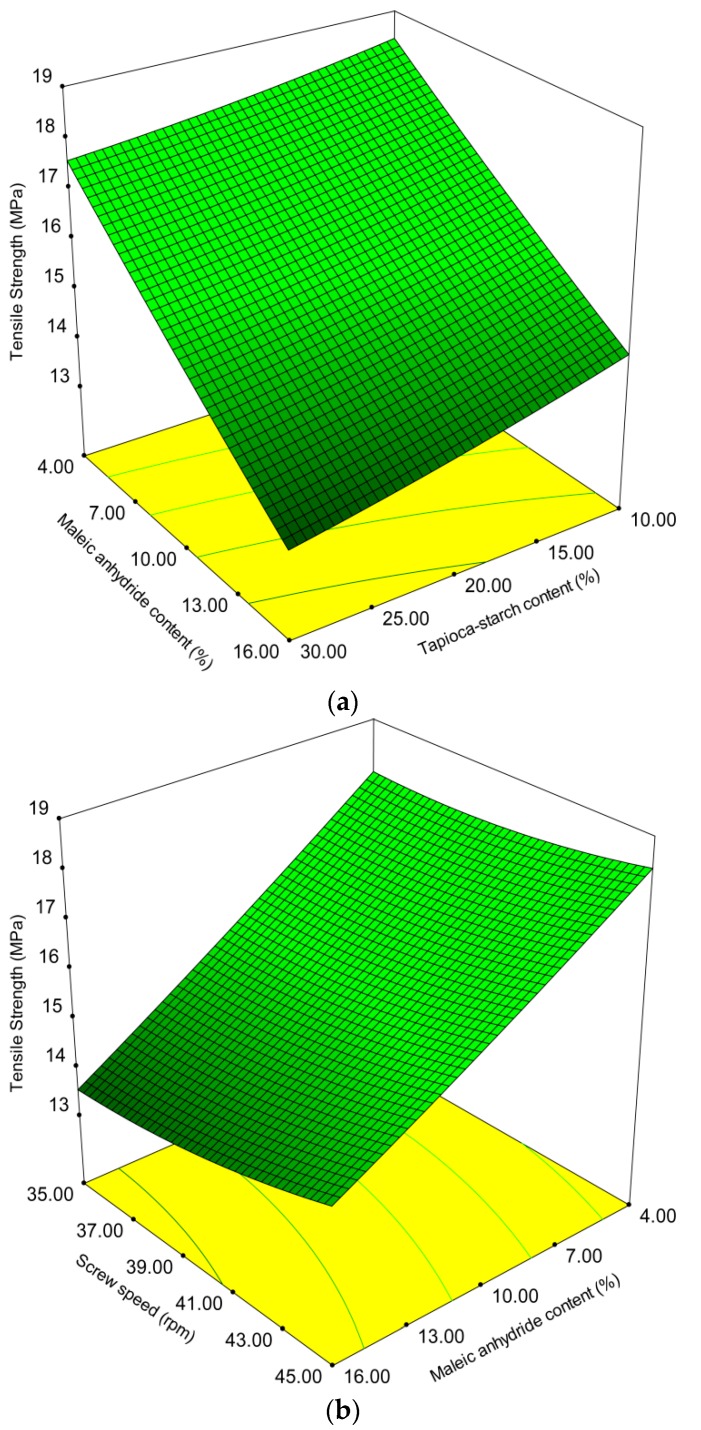
Effect of operating variables on tensile strength. (**a**) Tapioca starch content versus maleic anhydride content at a screw speed of 280 rpm; (**b**) maleic anhydride content versus screw speed level at 20% tapioca starch content.

As shown in Table 2, the results of the tensile strength measurements for various treatments ranged from 13.2 to 18.5 MPa. Table 3 shows that both the tapioca starch content and MA content have a negative linear effect on tensile strength. However, screw speed level and its square effect have a positive linear effect on tensile strength. This results in an increase in the tensile strength value because of faster moisture loss and the lack of penetration of heat with increasing screw speed. Moreover, the interaction of tapioca starch content and MA content as well as the interaction of tapioca starch content (*X*_1_) and screw speed level (*X*_3_) were found to have a negative effect on tensile strength. The presence of starch particles may not contribute to the mechanical properties of the composite due to starch molecules being easily depolymerized by heating or heated-sheared treatment [32,41]. The interaction of the MA content (*X*_2_) and screw speed level (*X*_3_) has a positive effect on tensile strength. Increasing screw speed decreases reaction time, thereby reducing molecular cleavage and the reaction between stress-induced macro radicals and MA. The decrease in tensile strength can be associated with extra MA molecules due to the presence of MA aggregates in the composite [42]. Kakou et al. reported that fiber-matrix interface saturation occurred with less than 4% MA in the matrix bulk [43]. That is, excess MA molecules would obstruct the ester bonds between the matrix and the MA function, and the crosslinking reaction was significantly suppressed. Similar results were obtained for other systems [30,44]. However, Yan et al. stated that the extruder exerted a mechanochemical reaction on the composite due to mechanical forces such as shearing force, friction, and extruding force, thereby reducing its mechanical properties [45]. Figure 1a,b, respectively, shows the effects of different tapioca starch content, MA content and screw speed level on tensile strength in 3D surface response and 2D contour plots. For any designated quantity of tapioca starch content from 10% to 30%, the tensile strength decreases proportionally with MA content. In contrast, the tensile strength increased when the tapioca starch content was reduced from 30% to 10% at any level of constant MA content from 4–16%.

### 3.3. Elongation at Break

**Figure 2:**
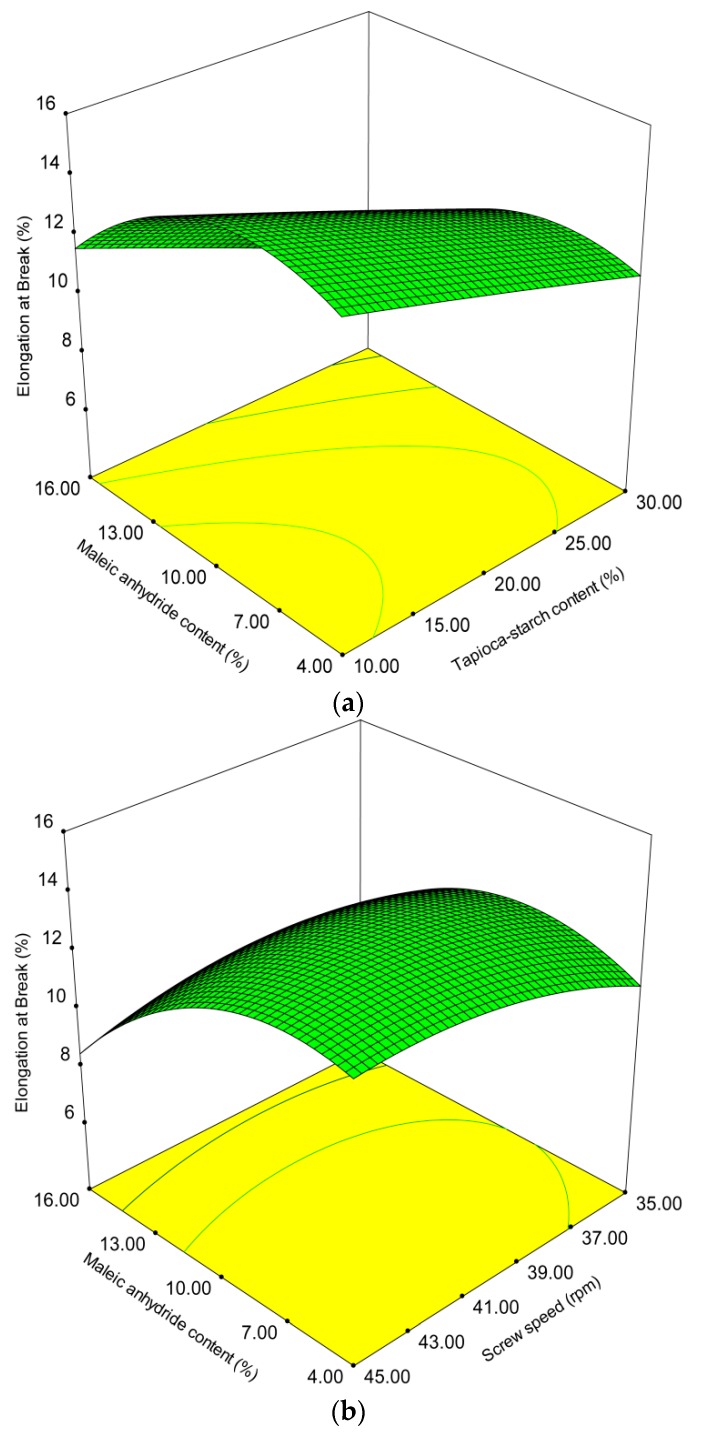
Effect of operating variables on elongation at break. (**a**) Tapioca starch content versus maleic anhydride content at a screw speed of 280 rpm; (**b**) maleic anhydride content versus screw speed level at 20% tapioca starch content.

The results of elongation at break measurements of each treatment group range from 7.5% to 14.2%, as shown in Table 2. Table 3 shows that both the tapioca starch content and MA content have a negative linear effect on elongation at break values. However, screw speed level has a positive linear effect on elongation. Increasing screw speed decreases reaction time, but also results in increased mixing in the extruder, which is in good agreement with previous work [46]. The interaction term of MA content and screw speed has a negative effect on melt processing [47]. The square effects on elongation are shown in terms of MA content (*X*_2_) and screw speed level (*X*_3_). Figure 2a,b, respectively, shows the effects of different levels of tapioca starch content, MA content, and screw speeds on elongation at break in 3D surface response and 2D contour plots. Figure 2b indicates that the MA content and screw speed level have a positive parabolic effect on the elongation values. Within the studied range of MA content, elongation at break increased as tapioca starch content was reduced. Roughly about 10% MA had a relatively higher elongation values. The hydroxyl groups of plasticizers and starch molecules would also interact with the compatibilizer while promoting interfacial adhesion [48]. In the present case, if maleation is assumed to not occur, then plasticizers should have increased elongation and interfacial adhesion in all blends, regardless of the composition of the compatibilizer. Ideally, the optimal compatibilized blend is a compromise between the desired elongation at break and MA content.

### 3.4. Water Absorption

**Figure 3:**
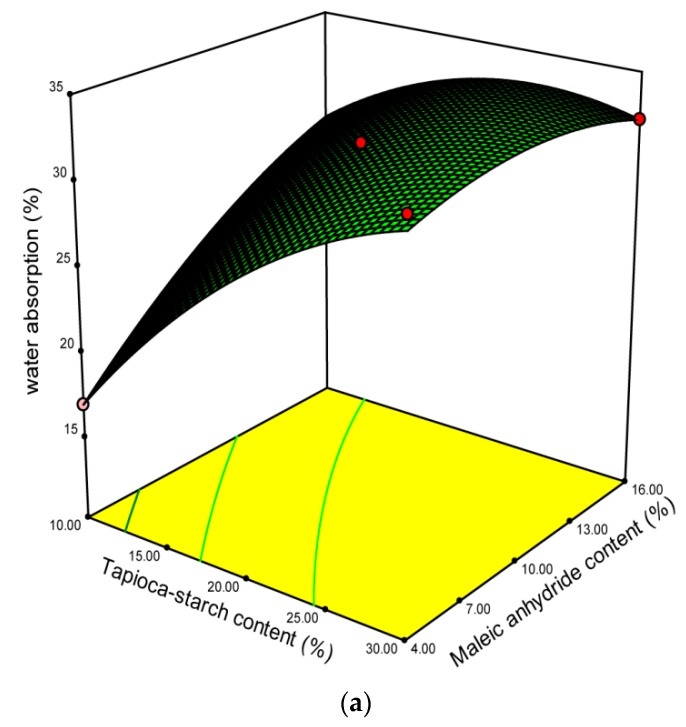

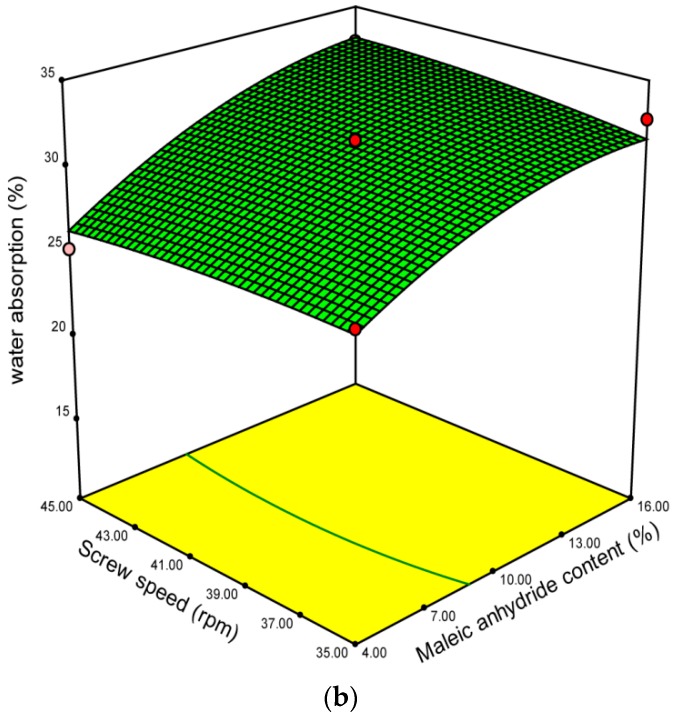
Effect of operating variables on water absorption. (**a**) Tapioca starch content versus maleic anhydride content at a screw speed of 280 rpm; (**b**) maleic anhydride content versus screw speed level at 20% tapioca starch content.

As shown in Table 2, the results of water absorption measurements for each treatment group ranged from 16.97% to 34.07%. Table 3 shows that both the tapioca starch content and MA content have a positive linear effect on water absorption. The interaction of tapioca starch content and MA content has a negative effect on water absorption. In addition, the tapioca starch content has a square effect on water absorption. Figure 3a,b, respectively, shows the effects of different tapioca starch content, MA content, and screw speed level on water absorption in 3D surface response and 2D contour plots. At a higher MA content of 16%, water absorption is only marginally affected by the tapioca starch content, while at lower MA content of 4%, water absorption significantly increased. Moreover, at any screw speed from 35 to 45 rpm, water absorption increases slightly with MA content. The greatest water absorption values may be due to the disintegration of starch granules and low molecular compounds from extrudate melt during the extrusion process and this may cause an increase in soluble material [49,50]. The improved interfacial adhesion could be caused by some chemical reaction between MA attached on the starch and the polymer matrix or by van der Waals forces between them under high temperature and high shear extrusion conditions [51].

### 3.5. Morphology of the Blends

**Figure 4:**
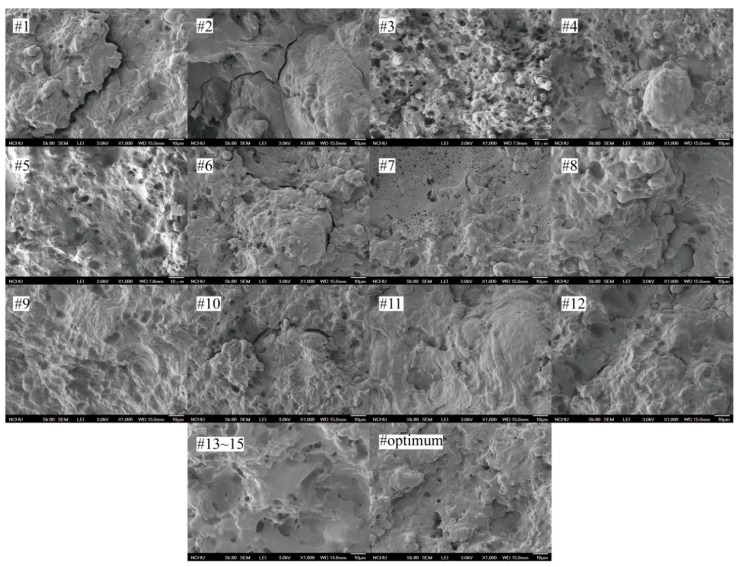
Micrographs of each treatment and optimized sample of PHBV/PVAc/tapioca starch blends at 1000× magnification. Blend descriptions are given in Table 2 and Table 4.

Figure 4 shows the micrographs of the fracture surfaces of selected extrudates (#1 to #15 and the optimum extrudate). The presence of the heterogeneous phase in the melt process affected the mechanical properties of the blends [52]. Although it was apparently hard to observe morphological features of the blends under the influence of extruder screw speed, extruder screw speed has a statistical effect on mechanical properties of the blends. Incorporating 4% of MA content, clear edges are visible between the starch granules and the PHBV/PVAc matrix indicating poor interfacial adhesion. The addition of about 10% of MA content to the blend has a noticeable effect on the composite morphology. This is possibly due to the improvement of interfacial adhesion between the phases. Hassaini et al. investigated the phase morphology of PHBV/biofiller blends with MA as a compatibilizer, and partial miscibility was found with the filler and the polymeric matrix [52]. Similar results were obtained by Ma et al. [53]. However, increasing content of MA to 16% produces large domain sizes of the round cavities in the blends. This not only resulted in a decrease in tensile strength of the samples, but also triggered catastrophic failure in elongation at the break values. Zhou et al. found that excessive MA content (over 12 wt %) in the polymeric matrix led to a decrease of the uniformity and interfacial adhesion between the filler and matrix by forming layers of macromolecules [54]. Moreover, as tapioca starch content increased, the surface became rough in terms of the increment of the number and size of agglomerates, resulting in the blends having poor mechanical properties. The micrographs of the central point group (#13~15) and optimum sample, the starch granules are well covered by the polymeric matrix. This could be attributed to appropriate MA content reducing the filler interactions and, furthermore, improving interfacial adhesion by the reaction of the anhydride group between the hydroxyl groups of PHBV/PVAc matrix and the tapioca starch.

### 3.6. Optimal and Model Verification

**Figure 5:**
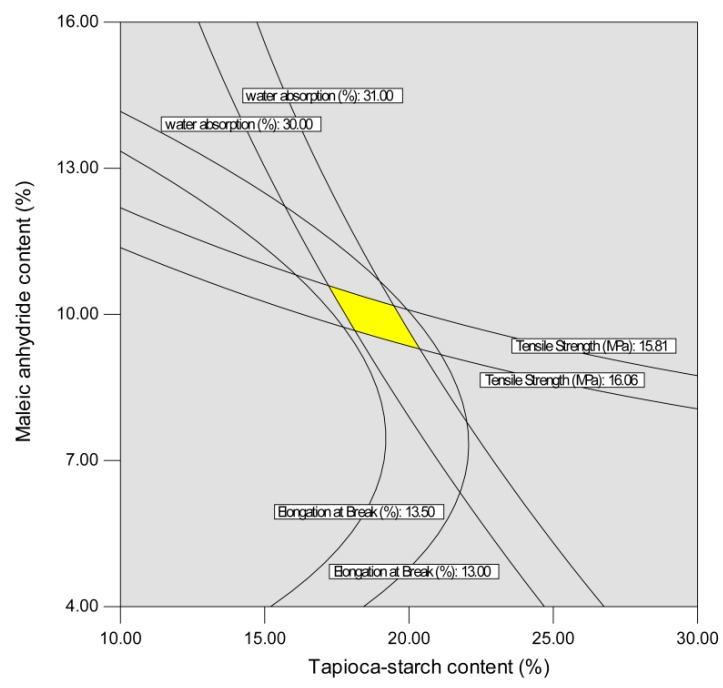
Superimposed contours for product responses affect by tapioca starch content and maleic anhydride content at a screw speed of 280 rpm.

**Figure 6:**
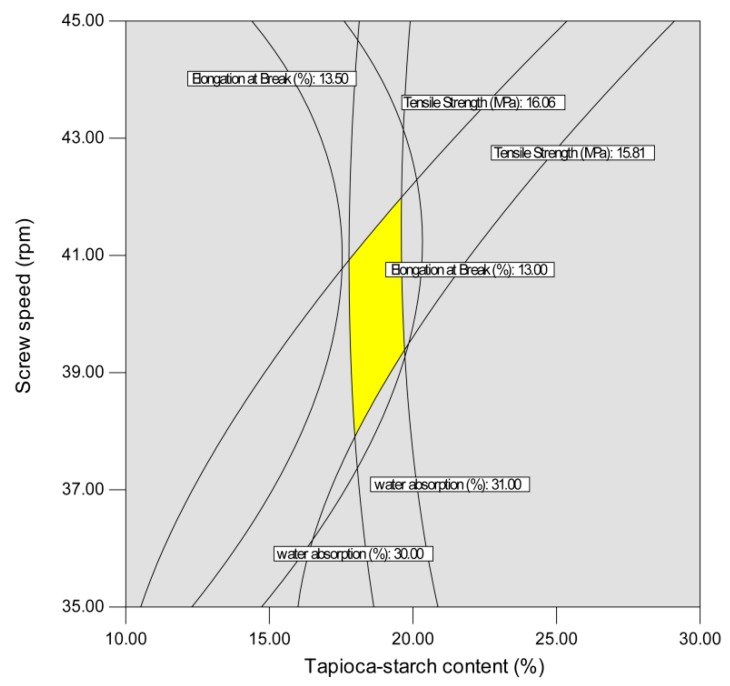
Superimposed contours for product responses affect by tapioca starch content and screw speed at 14% of maleic anhydride content.

The independent variables were optimized numerically using Design Expert version 7 (Stat-Ease Inc., Minneapolis, MN, USA) according to the experimental variables and regression analysis of the reaction between values. Areas of optimum performance were located by superimposing contour graphs for tensile strength, elongation at break, and water absorption for composition levels, which established limits of acceptable quality for each factor. While the optimum processing variables for each response did not fall exactly in the same region in the two-dimensional space formed by the composition levels, the constraints were set such that all responses fell within their optimum acceptable region with the same composition levels. The desired goals for each independent variable and response were chosen, as seen in Table 4. The independent variables were kept in range. The tensile strength and elongation at break were kept at maximum, and water absorption was kept at minimum. The overlapping of the contour plots presented in Figure 5 and Figure 6 accounts for the three responses for their optimal values corresponding to two variables at a time. The optimized operating range was as follows: 17.72% < tapioca starch content < 20.50%, 9.53% < MA content < 11.84%, 38.3 rpm < screw speed < 42.2 rpm. In the numerical analysis, the goals are combined into an overall desirability function [55]. Desirability is an objective function that ranges from zero (outside the desirability range) to one (at the goal). The conditions possessing the highest desirability value are selected as optimum values for the desired response. This type of methodology has been successfully applied for optimizing the preparation of biocomposites based on poly(lactic acid) and durian peel cellulose [56]. From the numerical analysis, 20.13% tapioca starch content, 10.14% MA content, and screw speed of 41.3 rpm gave an optimized product at a desirability of 0.83 by using the desirability approach to simultaneously optimize the responses. The model was tested experimentally by producing an extrudate under the predicted optimum conditions. To check the variety of predicted responses and the actual values, one sample *t*-test (two-tailed) was carried out (|*t* value| < 2.447). The actual values mentioned in Table 4 are the means of seven replicates with standard deviations. The actual values of tensile strength, elongation at break, and water absorption were respectively 16.38 MPa, 13.18%, and 30.87%, which are relatively close to the software generated values (16.4 MPa, 13.2%, and 30.94%). The *t*-test found no significant difference between the values of actual responses and the predicted responses and also showed that all the developed models were suitable for the optimum operating conditions.

## 4. Conclusions

Response surface methodology was successfully applied using the BBD. The responses of PHBV/PVAc/tapioca starch blended composite prepared through a single-screw extruder were affected by screw speed and compositional variables (tapioca starch and MA contents). These variables had some significant impacts on extrudate properties. Tapioca starch content and MA content significantly affected tensile strength, elongation at break, and water absorption values, while the extruder screw speed affected tensile strength. The treatments containing 10% MA had a relatively higher elongation. The interaction of tapioca starch content and MA content has a negative effect on water absorption. Through computerized simulations, the developed models and the resulting 3D surface response and 2D contour plots were found to be statistically valid and effective in simplifying the complexity of the preparation of processing conditions. An optimum operating range was determined by superimposing the contour plots of individual responses (tensile strength, elongation at break, and water absorption) as follows: tapioca starch content 17.72–20.50%, MA content 9.53–11.84%, and extruder screw speed 38.3–42.2 rpm.

## Figures and Tables

**Table 1:** Box-Behnken design matrix.

Treatments	Coded			Variables		
	*X*_1_	*X*_2_	*X*_3_	Tapioca Starch Content (%)	Maleic Anhydride Content (%)	Screw Speed (rpm)
1	−1	−1	0	10	4	40
2	1	−1	0	30	4	40
3	−1	1	0	10	16	40
4	1	1	0	30	16	40
5	−1	0	−1	10	10	35
6	1	0	−1	30	10	35
7	−1	0	1	10	10	45
8	1	0	1	30	10	45
9	0	−1	−1	20	4	35
10	0	1	−1	20	16	35
11	0	−1	1	20	4	45
12	0	1	1	20	16	45
13	0	0	0	20	10	40
14	0	0	0	20	10	40
15	0	0	0	20	10	40

**Table 2:** Response values for different experimental conditions.

Treatments	Variables			Responses		
	Tapioca Starch Content (%)	Maleic Anhydride Content (%)	Screw Speed (rpm)	Tensile Strength (MPa)	Elongation at Break (%)	Water Absorption (%)
1	10	4	40	18.5 ± 0.13	14.2 ± 0.25	16.97 ± 0.40
2	30	4	40	17.6 ± 0.15	10.9 ± 0.90	32.99 ± 0.36
3	10	16	40	14.6 ± 0.38	11.9 ± 0.40	27.51 ± 0.72
4	30	16	40	13.2 ± 0.17	7.5 ± 0.20	32.24 ± 1.19
5	10	10	35	16.0 ± 0.13	14.0 ± 0.75	23.61 ± 0.58
6	30	10	35	15.4 ± 0.28	9.9 ± 0.45	31.49 ± 0.54
7	10	10	45	17.3 ± 0.31	14.1 ± 0.60	24.59 ± 0.27
8	30	10	45	15.8 ± 0.12	10.9 ± 0.35	34.07 ± 0.20
9	20	4	35	17.9 ± 0.11	11.1 ± 0.20	27.20 ± 1.21
10	20	16	35	13.6 ± 0.50	8.8 ± 0.20	32.77 ± 0.69
11	20	4	45	18.3 ± 0.15	13.3 ± 0.20	25.26 ± 0.68
12	20	16	45	14.7 ± 0.29	8.4 ± 0.10	32.79 ± 0.09
13	20	10	40	15.9 ± 0.08	12.9 ± 0.05	31.65 ± 0.04
14	20	10	40	15.8 ± 0.07	13.0 ± 0.05	30.28 ± 0.12
15	20	10	40	15.8 ± 0.06	13.1 ± 0.05	31.58 ± 0.28

**Table 3:** Regression summaries and analysis of variance.

Variables	Tensile Strength (MPa)	Elongation at Break (%)	Water Absorption (%)
Model	Quadratic	Quadratic	Quadratic
**Intercept**	15.83 **	13.00 **	31.17 **
***X*_1_**	−0.55 **	−1.88 **	4.76 **
***X*_2_**	−2.03 **	−1.61 **	2.86 **
***X*_3_**	0.40 **	0.36 *	0.21
***X*_1_*X*_2_**	−0.12 *	−0.28	−2.82 **
***X*_1_*X*_3_**	−0.23 **	0.23	−0.40
***X*_2_*X*_3_**	0.17 *	−0.65 **	0.49
***X*_1_^2^**	0.07	−0.03	−2.40 *
***X*_2_^2^**	0.07	−1.85 **	−1.34
***X*_3_^2^**	0.22 **	−0.75 **	−0.33
**F-value (model)**	562.01 **	96.26 **	22.02 **
**CV%**	0.53	2.38	4.29
***R*^2^**	0.999	0.994	0.975
**Adjusted *R*^2^**	0.997	0.984	0.931
**Adeq. Precision**	73.655	30.886	16.555
**Lack-of-fit(*p*-value) ^ψ^**	0.259	0.077	0.221

* Indicates significant difference (*p* < 0.05). ** Indicates most significant difference (*p* < 0.01). ^ψ^ Selected model exhibits insignificant lack-of-fit (*p* > 0.05). *X*_1_: tapioca starch content (%); *X*_2_: maleic anhydride content (%); *X*_3_: screw speed (rpm).

**Table 4:** Optimum values of operating conditions and responses.

Variables	Target	Optimum Value	Desirability	Actual Value ^a^	*t* Value
tapioca starch content (%)	in range	20.13			
maleic anhydride content (%)	in range	10.14			
screw speed (rpm)	in range	41.3			
**Responses**		**Predicted values**	0.79		
tensile strength (MPa)	maximize	16.4		16.38 ± 0.13	−0.555
elongation at break (%)	maximize	13.2		13.18 ± 0.05	−1.25
water absorption (%)	minimize	30.94		30.87 ± 0.09	−0.543

$H_{0}:\;\mu_{0}=\;\mu_{1},\;t_{\mathrm{cal}}\;<t_{\mathrm{Actual}\;\mathrm{value}}$ at *p* < 0.05, $H_{0}$ was accepted. |*t* value| < 2.447. ^a^ Means of seven replications.
